# Physical and Psychological Burdens Among Breast Cancer Survivors: Evaluating Post-Treatment Gait Impairment, Falls, and Depression Using Real-World Data

**DOI:** 10.1155/ecc/5558563

**Published:** 2025-10-06

**Authors:** Asmaa Namoos, Nicholas Thomson, Carol Olson, Vanessa Sheppard, Michel Aboutanos

**Affiliations:** 1 Department of Surgery, School of Medicine, Virginia Commonwealth University, Richmond, Virginia, USA; 2 Department of Social and Behavioral Sciences, School of Public Health, Virginia Commonwealth University, Richmond, Virginia, USA

**Keywords:** anxiety, breast cancer survivors, depression, falls, gait abnormalities, mobility impairment, physical impairment, psychological distress, stress-related disorders, survivorship care

## Abstract

**Background:**

Breast cancer survivors face a dual burden of physical and psychological challenges, which may persist long after treatment. This study aims to evaluate the physical impairments and psychological outcomes among breast cancer survivors compared to individuals without breast cancer.

**Methods:**

We conducted a retrospective cohort study using data from 3650 breast cancer survivors and 145,280 individuals without breast cancer from the Virginia Commonwealth University Health System (VCUHS) between January 2024 and 2025. Data were extracted through the TriNetX platform using ICD-10 codes to identify relevant diagnoses and outcomes. Physical outcomes included abnormalities of gait and mobility, unsteadiness on feet, and falls. Psychological outcomes assessed were depression, stress-related disorders, and anxiety following falls. Risk differences, risk ratios (RR), and odds ratios with 95% confidence intervals (CIs) were calculated to compare outcomes between groups.

**Results:**

Breast cancer survivors exhibited a higher risk of physical impairments compared to nonbreast cancer individuals. The risk of gait and mobility abnormalities was 2.725% in breast cancer survivors versus 1.741% in the comparison group (RR: 1.565; 95% CI: 1.285–1.906; *p* < 0.0001). Unsteadiness on feet was more prevalent among breast cancer survivors (0.817%) compared to 0.296% in non-cancer individuals (RR: 2.762; 95% CI: 1.91–3.993; *p* < 0.0001). Additionally, breast cancer survivors had a higher risk of falls (1.644%) compared to nonbreast cancer patients (1.081%) (RR: 1.521; 95% CI: 1.178–1.964; *p* = 0.0012). Psychologically, breast cancer survivors who experienced falls were more likely to suffer from depression (28.57% vs. 13.1%, *p* = 0.0002), stress-related disorders (14.29% vs. 3.49%, *p* < 0.0001), and anxiety (28.57% vs. 15.72%, *p* = 0.0040) compared to fallers without breast cancer.

**Conclusion:**

Functional limitations such as unsteadiness and falls are significantly more common among breast cancer survivors and are strongly associated with psychological distress. These findings support mobility impairments as a potential pathway linking cancer treatment to adverse mental health outcomes. Future research should integrate structured ICD-10 data with unstructured oncology notes to enhance fall prediction models and guide personalized survivorship care.

## 1. Background

Breast cancer (BC) not only presents a significant physical threat to women but also induces profound emotional and psychological trauma [1, 2]. The diagnosis of BC is often experienced as a life-altering event, causing immediate distress and triggering a cascade of psychological reactions such as fear, anxiety, and grief [3]. Beyond the diagnosis itself, the treatment process, comprising surgery, chemotherapy, radiation, and hormone therapies, can impose lasting physical and psychological burdens [4]. These treatments are essential for survival but often come with serious side effects that impact both the body and mind [5].

The physical consequences of BC treatment can include chronic pain, lymphedema, changes in appearance (such as mastectomy or hair loss), and impaired mobility [6]. These side effects can severely affect daily functioning, leading to a decline in the quality of life. Additionally, treatments like chemotherapy and radiation can result in long-term damage to tissues and organs, contributing to a higher risk of physical impairments.

The physical impairments such as gait abnormalities, unsteadiness, and falls are likely to be a direct consequence of the long-term effects of cancer treatments such as chemotherapy [7, 8]. These treatments, while essential for survival, often lead to side effects that affect mobility and balance. For instance, chemotherapy can cause peripheral neuropathy, which impairs sensory perception in the limbs, leading to difficulties in walking and maintaining balance [9, 10]. Similarly, radiation can result in tissue damage and muscle weakness, which can further compromise mobility [11]. The increased risk of falls observed among cancer patients, as falls can lead to injuries that may exacerbate both physical and emotional distress, creating a cycle of decline.

The psychological challenges faced by cancer patients begin at the moment of diagnosis, even before the start of treatment [4]. Symptoms such as depression, anxiety, and stress are often intensified by the emotional and physical toll of the cancer journey, further compounded by the side effects of treatment.

Psychologically, the trauma of cancer treatment can persist long after the completion of therapy [12]. Many cancer survivors experience a heightened vulnerability to stress, anxiety, and depression, often stemming from the fear of cancer recurrence and the emotional toll of navigating life post-treatment [13]. For some, the trauma is compounded by the physical changes they endure, such as changes in body image or sexual function, which can exacerbate feelings of self-consciousness or loss [14]. Psychological conditions, including post-traumatic stress disorder (PTSD) and other stress-related disorders, may develop as survivors struggle to adjust to their “new normal.”

These physical and psychological challenges are intertwined, with one often amplifying the other. The cumulative impact of both the disease and its treatment forms a dual burden that hinders recovery, underscoring the importance of comprehensive survivorship care. However, despite increasing recognition of these challenges, there remains a gap in research that explores how these outcomes manifest and interact over time, particularly in relation to falls and their subsequent psychological effects.

This retrospective study, conducted using data from the TriNetX platform at Virginia Commonwealth University Health System (VCUHS), aims to investigate the dual burden of trauma experienced by BC survivors 1 year after diagnosis, focusing on both the physical impairments and psychological consequences following cancer treatment. The study compares BC survivors to individuals who visited VCUHS during 2024–2025 to assess the incidence and prevalence of falls, as well as the associated physical conditions such as abnormalities in gait, mobility issues, and unsteadiness. Additionally, the study explores the psychological impacts, including depression, anxiety, and stress-related disorders, which may be exacerbated by the trauma of cancer treatment and the experience of falls.

## 2. Methods

### 2.1. Study Design.

This retrospective study includes 3650 BC patients who visited the VCUHS between January 2024 and 2025. The study utilizes electronic health records (EHRs) from the TriNetX network at VCUHS drawing on detailed demographic and clinical data to examine physical and psychological outcomes following a BC diagnosis.

### 2.2. Patient Selection.

Patients were selected based on the occurrence of specific outcomes following their BC diagnosis. The study focused on evaluating the incidence of physical and psychological conditions that developed after the diagnosis of BC (Stage I-III) within 1year. These outcomes included abnormalities of gait and mobility, unsteadiness on feet, falls, depression, stress-related disorders, and anxiety.

### 2.3. International Classification of Diseases (ICD-10) Codes Used in the Study.

The identification of clinical outcomes was conducted using ICD-10 codes based on the World Health Organization’s (WHO). These codes were used to capture various physical and psychological conditions relevant to the study. The specific ICD-10 codes and corresponding diagnoses are presented in Table 1:

### 2.4. Data Source.

Per TriNetX platform, is an extensive health research network that links healthcare organizations with life sciences companies, offering real-world data to support clinical research and the discovery of new treatments. The platform grants researchers access to anonymized and aggregated EHR data, allowing for observational studies, cohort comparisons, and the generation of evidence-based insights to improve healthcare outcomes [15].

### 2.5. Data Collection.

Data were extracted from the EHRs available through the TriNetX network at VCUHS. The dataset includes detailed demographic information, such as age, sex, race, and ethnicity, along with ICD-10 diagnosis codes [16]. Additionally, it records information on falls, recurrence of falls, types of depression. The dataset also captures the timing and severity of falls, hospital visits related to these incidents, and clinical history relevant to fall risk factors.

### 2.6. Handling of Multiple Records and Missing Data.

In this study, each patient was analyzed individually. If a patient had multiple fall incidents during the study period, only the first recorded fall was included in the primary analysis. Any additional falls that occurred more than 1 year after the initial incident were documented as recurrent falls but were not treated as distinct events for analysis purposes.

The TriNetX platform ensures high standards of data completeness and quality. Clinical variables are encoded as binary indicators (1 = present, 0 = absent), and null values are automatically excluded from cohort calculations. As a result, there were no missing values for key variables such as diagnosis codes or demographic information, and no data imputation was required [15].

### 2.7. Statistical Analysis.

All analyses were conducted using the built-in tools within the TriNetX platform. Descriptive statistics were generated to summarize demographic characteristics of both cohorts, including age, sex, and race. Continuous variables were summarized using means and standard deviations, while categorical variables were presented as frequencies and percentages.

To compare outcomes between BC survivors and individuals without BC, risk differences, risk ratios (RR), and odds ratio (OR) were automatically calculated by the platform, each with 95% confidence intervals (CIs). These measures were used to assess physical outcomes (gait abnormalities and unsteadiness, falls) and psychological outcomes (depression, anxiety, and stress-related disorders).

Two-proportion z-tests were used to compare binary outcomes between cohorts. Where applicable, Pearson’s chi-square tests were performed to assess the statistical significance of associations between categorical variables. A *p* value of less than 0.05 was considered statistically significant.

### 2.8. SoftwareandTools.

All analyses were conducted using the TriNetX platform’s analytical tools. These tools enabled processing of large datasets and provided robust statistical evaluations and visualizations to support epidemiological analysis.

## 3. Results

### 3.1. Demographic Characteristics.

The study included a total of 3650 BC survivors who have visited the VCUHS, with participants ranging in age from 24 to 89 years. The mean age of the cohort was 65 years, with a standard deviation of 12 years, reflecting a diverse age distribution. The age distribution showed a gradual increase in patient numbers from the mid-40s, peaking around the age of 70, and then tapering off towards the upper age limits.

All participants in the cohort were female, consistent with the focus on BC survivors. Regarding race, the majority of the participants identified as White (60.27%), followed by Black or African American individuals, who constituted 31.50% of the cohort. Other racial groups represented smaller proportions, including individuals classified as Other Race (2.73%), Asian (1.91%), American Indian or Alaska Native (0.27%), and Native Hawaiian or Other Pacific Islander (0.27%). A small percentage of the cohort had an unknown race classification (3.01%).

Ethnicity data were not available for this cohort, as 100% of the participants had unknown ethnicity status. The absence of ethnicity data limits the ability to explore potential differences related to Hispanic or Latino heritage in this analysis (Table 2).

### 3.2. Physical Impact of BC

#### 3.2.1. Abnormalities of Gait and Mobility.

The analysis of gait and mobility abnormalities between BC patients and individuals without BC (non-BC) revealed significant differences. In the BC cohort (*N* = 3650), 100 patients (2.725%) exhibited abnormalities in gait and mobility. In comparison, 2530 individuals (1.741%) in the non-BC cohort (*N* = 145,280) were affected by similar issues. The risk difference between these two groups was 0.983% (95% CI: 0.452%–1.514%), with the BC group showing a notably higher risk. The calculated RR was 1.565 (95% CI: 1.285–1.906), and the OR was 1.58 (95% CI: 1.291–1.935), both statistically significant (*p* < 0.0001). These results indicate that BC patients are approximately 1.56 times more likely to experience abnormalities in gait and mobility compared to non-BC patients, emphasizing the potential impact of BC and its treatments on physical function (Table 3).

#### 3.2.2. Gait Instability (Unsteadiness on Feet).

A similar pattern was observed when analyzing the prevalence of unsteadiness on feet. In the BC cohort, 30 individuals (0.817%) reported unsteadiness, while 430 individuals (0.296%) in the non-BC cohort experienced the same issue. The risk difference between these two groups was 0.521% (95% CI: 0.229%–0.814%), indicating a significantly higher risk of unsteadiness among BC patients. The RR for this outcome was 2.762 (95% CI: 1.91–3.993), and the OR was 2.776 (95% CI: 1.915–4.026), both of which were statistically significant (*p* < 0.0001). These findings further highlight that BC patients are nearly 2.8 times more likely to experience unsteadiness on their feet compared to non-BC individuals (Table 3).

#### 3.2.3. Falls.

In terms of falls, the BC cohort also showed a significantly higher risk. Among the 3650 BC patients, 60 (1.644%) experienced a fall. In contrast, within the non-BC cohort (*N* = 145,280), 1570 individuals (1.081%) fell. The risk difference between these two groups was 0.563% (95% CI: 0.147%–0.979%), indicating a higher likelihood of falls in BC patients. Statistical analysis revealed a z-score of 3.229, with a *p* value of 0.0012, confirming the statistical significance of this difference. The RR for falls in BC patients was 1.521 (95% CI: 1.178–1.964), and the OR was 1.53 (95% CI: 1.179–1.984), indicating that BC patients are about 1.5 times more likely to experience a fall compared to non-BC patients (Table 3).

### 3.3. Psychological Impact of BC

#### 3.3.1. Depression.

Among the BC cohort (*N* = 3650), 200 individuals (5.48%) experienced a depressive episode, compared to 7740 individuals (5.28%) in the non-BC cohort (*N* = 145,280). The risk difference was 0.20% (95% CI: 0.12%–0.29%), indicating a slightly higher prevalence of depression in the BC group. The RR was 1.041 (95% CI: 1.025–1.058), and the OR was 1.048 (95% CI: 1.030–1.066), with a *p* value of 0.0001, confirming a statistically significant association (Table 4).

#### 3.3.2. Stress-Related Outcomes.

When examining stress-related outcomes, including acute stress reactions and other stress-related disorders, a higher prevalence was observed among BC patients. Among the 3650 BC patients, 10 (0.27%) experienced stress-related conditions, compared to 120 (0.08%) in the non-BC cohort (*N* = 145,280). The risk difference between the two groups was 0.19% (95% CI: 0.02%–0.35%), with a RR of 3.305 (95% CI:1.732–6.363) and an OR of 3.305 (95% CI: 1.732–6.363), both of which were statistically significant (*p* < 0.0001) (Table 4).

#### 3.3.3. Anxiety.

Anxiety was significantly more prevalent among BC patients compared to individuals without BC. In the BC cohort, 400 individuals (10.89%) experienced anxiety, while 12,880 individuals (8.86%) in the non-BC cohort had anxiety-related disorders. The risk difference was 2.03% (95% CI: 1.01%–3.02%), with a RR of 1.229 (95% CI: 1.137–1.329) and an OR of 1.237 (95% CI:1.137–1.329), both statistically significant (*p* = 0.0001) (Table 4).

## 4. Discussion

This study aimed to explore the dual burden of trauma after 1 year faced by BC survivors, focusing on the physical and psychological challenges that persist beyond treatment. The findings from our analysis show that BC survivors experience a significantly higher incidence of physical impairments, such as gait abnormalities, unsteadiness on their feet, and falls, when compared to individuals without BC. Moreover, these survivors are more likely to suffer from psychological distress, including depression, anxiety, and stress-related disorders. Specifically, survivors who experienced falls reported elevated rates of these psychological conditions. The results underscore the intertwined nature of physical and psychological health in BC survivorship, suggesting that these survivors face compounded difficulties that affect both their bodies and minds.

In comparing our results to existing literature, our findings align with the body of research that documents the lasting physical and psychological consequences of BC treatment. Previous studies have shown that BC survivors are at an increased risk of physical impairments, including gait disturbances and falls. For example, Hsieh et al. conducted a systematic review and found that BC survivors frequently experience gait and balance deficits, which are associated with a higher risk of falls [8]. Similarly, the National Cancer Institute highlighted that chemotherapy and radiation treatments contribute to musculoskeletal weakness and neuropathy, both of which are known to impair mobility and increase fall risk [17, 18].

In our study, anxiety was the most prevalent psychological condition among BC patients, affecting 10.89% of the cohort. This was followed by depression at 5.48% and stress-related disorders at 0.27%. These findings are consistent with existing literature, which reports higher rates of anxiety and depression among BC patients compared to the general population. For instance, a study found that 38% of Chinese adult BC patients experienced both anxiety and depression [19]. Another study reported that 31.7% of BC patients experienced anxiety, while 22.0% experienced depression [20]. The lower prevalence rates in our study may be attributed to differences in sample size, assessment methods, or cultural factors. Also, the hormonal therapy may influence the patient’s mood, causing depression, while chemotherapy and antiemetic medications can contribute to increased anxiety levels [21, 22]. Furthermore, our results suggest that the physical challenges of BC survivorship—such as mobility impairments and fall risks—are not only detrimental in themselves but may also exacerbate psychological distress. This interconnectedness highlights the need for a more integrated approach to care that addresses both physical rehabilitation and psychological support.

### 4.1. Limitations.

While this study offers important insights, there are some limitations to consider. Since it is based on EHRs, the data depend on how accurately and consistently conditions were documented by healthcare providers. Another challenge is the presence of unmeasured confounders—factors like socioeconomic status, healthcare access, or prior lifestyle habits that were not accounted for but could have influenced both physical and psychological outcomes. Additionally, the study only followed patients for 1 year after diagnosis, leaving questions about how these issues evolve over time. Future research should take a longer-term and more comprehensive approach to capture these complexities.

### 4.2. Strengths.

Despite these limitations, this study provides meaningful insights into the challenges BC survivors face beyond treatment. By excluding common health conditions such as hypertension, other chronic diseases, and pre-existing mental health disorders, we ensured a more focused assessment of the direct impact of BC and its treatment on physical and psychological well-being. Additionally, medication effects, such as antihypertensive drugs or psychotropic medications, were controlled, eliminating potential confounding influences on balance and mental health outcomes. By using real-world clinical data from a large health system, we were able to examine a sizable patient population, strengthening the reliability of our findings. A key strength of this study is that it highlights the connection between mobility issues and mental health struggles, reinforcing the need for survivorship care that addresses both body and mind. Our findings contribute to the growing recognition that cancer recovery requires more than just medical treatment—it calls for comprehensive, multidisciplinary support that improves survivors’ overall quality of life.

## 5. Conclusion

Our findings demonstrate that BC survivors are more likely to experience functional impairments—such as gait abnormalities, unsteadiness, and falls—compared to individuals without cancer. These impairments are clinically significant and are strongly associated with higher rates of depression, anxiety, and stress-related disorders, particularly among survivors who experience falls. This association suggests that mobility limitations may directly contribute to psychological distress, highlighting the need for survivorship care programs that simultaneously address physical and mental health.

To address this gap, future research should develop and evaluate interventions that proactively identify survivors at increased risk for falls and related psychological outcomes. One of our proposed next steps involves integrating structured data (e.g., ICD-10 codes) with unstructured clinical notes to improve predictive models for fall risk. This approach will support the development of personalized care pathways, including hospital-based programs grounded in Social Cognitive Theory (SCT). By incorporating guided physical therapy, cognitive reframing, and peer support, these programs aim to improve balance, restore confidence, and reduce anxiety. Embedding such interventions into clinical workflows may facilitate early engagement and promote long-term recovery for older adult BC survivors.

## Figures and Tables

**Table 1:** International classification of diseases (ICD-10) codes.

ICD-10 code	Diagnosis	Description

C50	Malignant neoplasm of breast	Breast cancer affecting glandular breast tissue
R26	Abnormalities of gait and mobility	Abnormalities of gait and mobility
R26.81	Unsteadiness on feet	Unsteadiness on feet
W19	Fall, unspecified	Unspecified fall
F43.0	Acute stress reaction	Severe stress response occurring shortly after a traumatic event
F40-F48	Neurotic, stress-related, and somatoform disorders	Includes phobic anxiety disorders, anxiety disorders, dissociative disorders, and somatoform disorders
F32	Depressive episode	Episodes of depressive mood with varying severity

**Table 2:** Demographic characteristics of breast cancer survivor.

Variable	Category	%	*n*

Sex	Female	100.0	3650

	Unknown Ethnicity	100.0	3650
Ethnicity	Hispanic or Latino	0.0	0
	Not Hispanic or Latino	0.0	0

	White	60.27	2200
	Black or African American	31.50	1150
	Unknown Race	3.01	110
Race	Other Race	2.73	100
	Asian	1.91	70
	American Indian or Alaska Native	0.27	10
	Native Hawaiian or other Pacific Islander	0.27	10

**Table 3:** Physical impact of breast cancer—comparative analysis of gait abnormalities, gait instability, and falls.

Condition	BC cohort (*N* =3650)	Non-BC cohort (*N* =145,280)	Risk difference (%)	Risk ratio (RR)	Odds ratio (OR)	*p* value

Abnormalities of Gait and mobility	100 (2.725%)	2530 (1.741%)	0.983% (95% CI: 0.452%–1.514%)	1.565 (95% CI: 1.285–1.906)	1.58 (95% CI: 1.291–1.935)	< 0.0001
Gait Instability (unsteadiness on feet)	30 (0.817%)	430 (0.296%)	0.521% (95% CI: 0.229%–0.814%)	2.762 (95% CI: 1.91–3.993)	2.776 (95% CI: 1.915–4.026)	< 0.0001
Falls	60 (1.644%)	1570 (1.081%)	0.563% (95% CI: 0.147%–0.979%)	1.521 (95% CI: 1.178–1.964)	1.53 (95% CI: 1.179–1.984)	0.0012

**Table 4:** Psychological impact of breast cancer—comparative analysis of depression, stress-related outcomes, and anxiety.

Condition	BC cohort (*N* = 3650)	Non-BC cohort (*N* = 145,280)	Risk difference (%)	Risk ratio (RR)	Odds ratio (OR)	*p* value

Depression	200 (5.48%)	7740 (5.28%)	0.20 (95% CI: 0.12–0.29)	1.04 (95% CI: 1.02–1.05)	1.048 (95% CI: 1.030–1.066)	0.0001
Stress-related outcomes	10 (0.27%)	120 (0.08%)	0.19 (95% CI: 0.02–0.35)	3.305 (95% CI: 1.732–6.363)	3.305 (95% CI: 1.732–6.363)	< 0.0001
Anxiety	400 (10.89%)	12,880 (8.86%)	2.03 (95% CI: 1.01–3.02)	1.229 (95% CI: 1.137–1.329)	1.237 (95% CI: 1.137–1.329)	0.0001

## Data Availability

The datasets generated and/or analyzed during the current study were extracted from the TriNetX database. These datasets are available from the corresponding author on reasonable requests, subject to privacy and ethical restrictions.

## Nomenclature

VCUHS: Virginia Commonwealth University Health System
ICD-10: International Classification of Diseases, 10th Revision
EHR: Electronic Health Records
